# Expansion of Molecular and Clinical Aspects of *EPS8L2* (DFNB106)-Associated Hearing Loss Emphasizes a Potential Therapeutic Window

**DOI:** 10.1007/s12035-025-05615-9

**Published:** 2026-01-10

**Authors:** Daniel Owrang, Aboulfazl Rad, Masoome Alerasool, Susanne M. Kolb, Sheng-Jia Lin, Julia Doll, Neda Alidadiani, Shahrooz Ghaderi, Michaela A. H. Hofrichter, Reza Maroofian, Gaurav K. Varshney, Majid Mojarrad, Oliver Bartsch, Thomas Haaf, Barbara Vona

**Affiliations:** 1 Institute for Auditory Neuroscience and InnerEarLab, University Medical Center Göttingen, 37075 Göttingen, Germany; 2 Auditory Neuroscience and Optogenetics Laboratory, German Primate Center, 37077 Göttingen, Germany; 3 Cellular and Molecular Research Center, Sabzevar University of Medical Sciences, Sabzevar, 009851 Iran; 4 Faculty of Medicine, Department of Medical Genetics, Mashhad University of Medical Sciences, Mashhad, Iran; 5 Medical Genetics Research Center, Mashhad University of Medical Sciences, Mashhad, Iran; 6 Genetic Laboratory, Genetic Foundation of Khorasan Razavi, Mashhad, Iran; 7 Institute of Human Genetics, Julius Maximilians University of Würzburg, 97074 Würzburg, Germany; 8 Genes & Human Disease Research Program, Oklahoma Medical Research Foundation, Oklahoma City, OK 73104 USA; 9 Institute of Pathology, University of Würzburg, Würzburg, Germany; 10 Atrak Biotech, Research & Development Division, Bojnurd, Iran; 11 Department of Neuromuscular Diseases, UCL Queen Square Institute of Neurology, London, WC1N 3BG UK; 12 Medical Care Centre Section Human Genetics and Institute of Human Genetics, University Medical Centre of the Johannes Gutenberg University Mainz, 55131 Mainz, Germany; 13 Collaborative Research Center 1690 (CRC1690), University of Göttingen, 37077 Göttingen, Germany; 14 Department of Obstetrics and Gynecology, Brigham and Women’s Hospital, Harvard Medical School, Boston, MA USA; 15 Program in Medical and Population Genetics, Broad Institute of MIT and Harvard, Cambridge, MA USA

**Keywords:** DFNB106, EPS8L2, Gene therapy, Hearing loss, Progressive hearing loss, Therapeutic window

## Abstract

*EPS8L2,* encoding EPS8 signaling adaptor L2 protein, is critical for stereocilia maintenance in cochlear hair cells. Four previously published families have replicated autosomal recessive non-syndromic hearing loss (DFNB106), yet the mutational and clinical spectrum remains poorly described. This study expands the mutational and clinical spectrum of *EPS8L2*-associated hearing impairment by identifying five additional individuals in four families, more than doubling the number of reported cases to date, while going deeper into clinical aspects. These include two frameshift variants c.818_827dup, p.(Ala279Glyfs*36) and c.1430dup, p.(Val478Serfs*25), the first nonsense variant c.1878C > A, p.(Tyr626Ter), and the first missense variant with a spliceogenic effect c.767C > G, p.(Thr256Arg). Functional analysis confirmed that the c.767C > G variant causes exon 9 skipping, leading to a frameshift r.701_768del, p.(Gly234Alafs*55). A shared 4.17 Mb homozygous region among two unrelated Iranian families with this variant suggested a founder effect. These individuals uniquely showed U-shaped audiograms as a new association of more severe middle frequency hearing loss. Clinically, affected individuals presented moderate to moderately severe hearing loss that was mostly progressive, with an onset starting between the prelingual stage, extending postlingually. A younger sibling of the first European proband diagnosed with DFNB106 passed newborn hearing screening but was identified with compound heterozygous variants, underscoring the importance of early genetic testing. The progressive nature of *EPS8L2*-related hearing loss is unusual for recessive non-syndromic forms and highlights the need for early monitoring and intervention. This work emphasizes the need for early molecular diagnosis and long-term clinical follow-up, while describing the natural history of *EPS8L2* that will be important for future therapeutic development.

**Supplementary Information:**

The online version contains supplementary material available at 10.1007/s12035-025-05615-9.

## Introduction

Genes essential for hearing play critical roles in the molecular machinery, development, or maintenance of outer and inner hair cells (OHCs and IHCs) of the organ of Corti. The OHCs and IHCs are comprised of actin-rich stereocilia bundles that are essential for the conversion of mechanical sound into electrical signals [1]. Hearing depends on mechanotransduction, a process that converts mechanical stimuli into electrical signals, and happens through activation of mechanically gated ion channels near the tips of the stereocilia [2]. IHCs transmit these signals to the brain, while OHCs amplify sound stimuli through electromotility. Proper stereocilia organization is essential for both cell types, yet the molecular composition of these essential structures is poorly understood, and genes that are regarded as important and involved in these processes require further research.

EPS8L2, a 715-amino acid actin-binding protein with 21 exons, belongs to the EPS8 protein family, which includes EPS8 and three EPS8-like proteins (EPS8L1–L3) with overlapping roles [3]. EPS8L2 localizes to stereocilia tips in cochlear and vestibular hair cells in the mouse inner ear. An *Eps8l2* knockout mouse model exhibited progressive hearing loss due to disorganization of hair bundles. These hair bundles showed abnormally shorter and fewer tall stereocilia, while middle and small rows remained unaffected [4]. Unlike EPS8L2, which maintains mature stereocilia, EPS8 is critical for their initial elongation, with variants in *EPS8* causing congenital deafness in mice and humans (DFNB102, OMIM 615974) [4–7]. *EPS8L2* (DFNB106, OMIM 614988) has been identified as an autosomal recessive non-syndromic hearing loss-associated gene in humans as well, mirroring the progressive hearing loss in mouse models [4, 8]. In humans, *EPS8L2*-associated hearing loss has so far shown a prelingual onset with high frequencies most severely affected [8–10].

A human genetics approach to unravel gene-disease function is a powerful way to identify genes and clinically characterize the effects of their disruption [11]. Despite the broad use of exome and genome sequencing, many genes that have been identified and implicated as important in normal hearing still remain grossly undercharacterized both from mutational spectrum as well as clinical course [11]. *EPS8L2* is one such gene, with only four families so far reported. Open questions remain with respect to the precise age of onset that has previously ranged from prelingual to 6 years of age and further replication of the two previously reported patients with progression of hearing loss [8, 10]. Both of these aspects are likely to impact tractability to preclinical models for eventual gene therapy trials.

We present five affected individuals from four families with *EPS8L2*-associated hearing impairment with clinical information, substantially contributing to the limited number of cases and alleles currently reported. We also characterize the first missense variant in *EPS8L2* and uncovered aberrant splicing, aligned with loss-of-function variants involved in the pathogenesis of DFNB106.

## Results

### Clinical Genetics and Variant Identification

#### Family 1

**Fig. 1:**
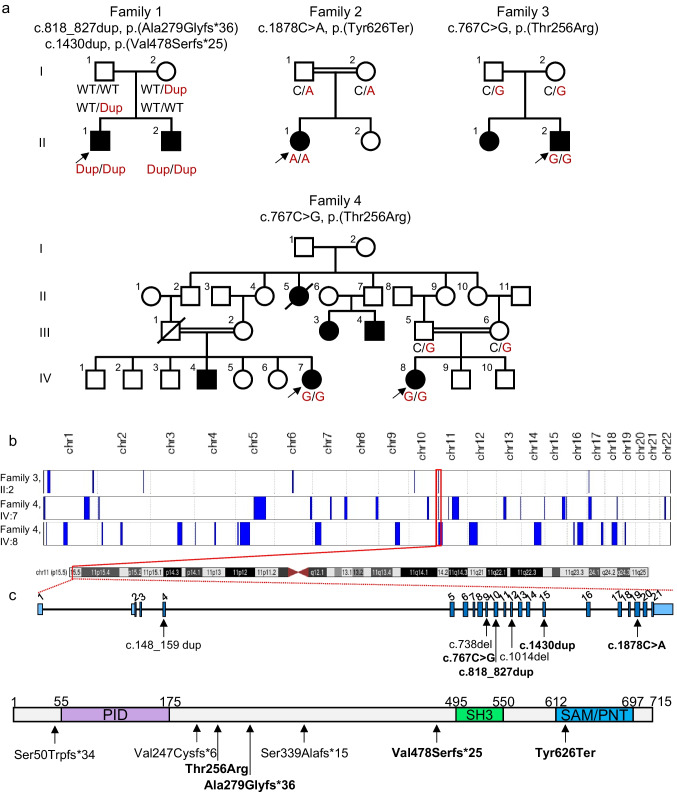
Pedigrees, autozygosity mapping, and map of *EPS8L2* variants on gene and protein level. **a** Pedigrees with segregation of *EPS8L2* variants. Arrows indicate the probands. The two affected individuals who were exome sequenced in Family 4 were originally regarded as two separate families, but later disclosure of distant family relationships was clarified, explaining why we regarded there to be two probands in the large extended family. **b** Autozygosity mapping using exome sequencing data of the proband in Family 3 and two distantly related affected individuals in Family 4 marked with an arrow. The common region of autozygosity was identified in chromosome 11 that contains *EPS8L2* and is boxed in red. **c** Schematic presentation of all *EPS8L2* variants described to date with the variants in the families we present marked in bold on gene- (NM_022772.4, upper panel) and protein-levels (NP_073609.2, lower panel). EPS8L2 contains a phosphotyrosine interaction domain (PID), a SRC Homology 3 (SH3) domain, and a sterile alpha motif/pointed domain (SAM/PNT)

The proband in family 1 was derived from non-consanguineous German parents (mother from Northern Greece, father from Germany) with no previous history of hearing loss and was ascertained as part of routine diagnostics at the Institute of Human Genetics, University Medical Centre of the Johannes Gutenberg University Mainz (Fig. 1A). He underwent newborn hearing screening and was tested twice with transitory evoked otoacoustic emission and automated auditory brainstem response with an abnormal result on the left ear and unremarkable result on the right ear. Retrospectively, from 2 years onwards, the parents had noted unclear language and that their son did not respond to sounds such as the doorbell. At the age of 3.5 years, the parents followed up with otolaryngology who performed the audiological testing. At the age of 4 years, the proband was diagnosed with bilateral sensorineural hearing loss (60 dB at 1 kHz bilaterally, 95 dB [left] and 100 dB [right] at 8 kHz) and was referred for genetic testing. The proband currently uses hearing aids.

Exome sequencing and segregation analysis uncovered a maternally inherited c.818_827dup, p.(Ala279Glyfs*36) in exon 10 out of 21 exons and paternally inherited c.1430dup, p.(Val478Serfs*25) variant in exon 15, conferring compound heterozygosity of pathogenic *EPS8L2* variants (NM_022772.4) (Fig. 1C). The c.818_827dup, p.(Ala279Glyfs*36) variant is absent from gnomAD v4.1.0, as well as the All of Us and TopMed v8 browsers and is, therefore, a new pathogenic disease-associated allele (PVS1_VS, PM2_P, PM3_M), while the c.1430dup, p.(Val478Serfs*25) pathogenic variant (PVS1_VS, PM2_P, PM3_S, PP1_P) has a population maximum minor allele frequency in gnomAD v4.1.0 of 1.9e-4 and has been classified as pathogenic in ClinVar (variation ID 1334121) and as a variant of unknown significance (VUS) in the Deafness Variation Database (DVD) (Table 1). It was previously identified in a homozygous state in a proband with profound, progressive hearing loss with an onset around 6 years of age [10]. Both variants are predicted to cause loss-of-function (Fig. 1C).

The proband has a younger brother who passed newborn hearing screening and was clinically normal at the age of 1 year. Predictive genetic testing of the *EPS8L2* variants showed compound heterozygosity. At 21.5 months, genetic counseling emphasized the need for close monitoring, and the parents reported the first signs of hearing difficulty around 23 months of age. At a telephonic follow-up at age 27 months, the father reported middle ear effusion, and although initial uncertainty remained regarding the contribution of effusion versus genetic predisposition, confirmatory audiograms at 2 years and 6 months demonstrated definitive hearing impairment. The child is now scheduled to receive his first hearing aids at 2 years and 8 months. To our knowledge, this represents the first report of an *EPS8L2* diagnosis in a family with European genetic ancestry and the first documentation of a pre-symptomatic individual in whom hearing loss developed following an initially normal newborn screening result.

#### Family 2

**Fig. 2:**
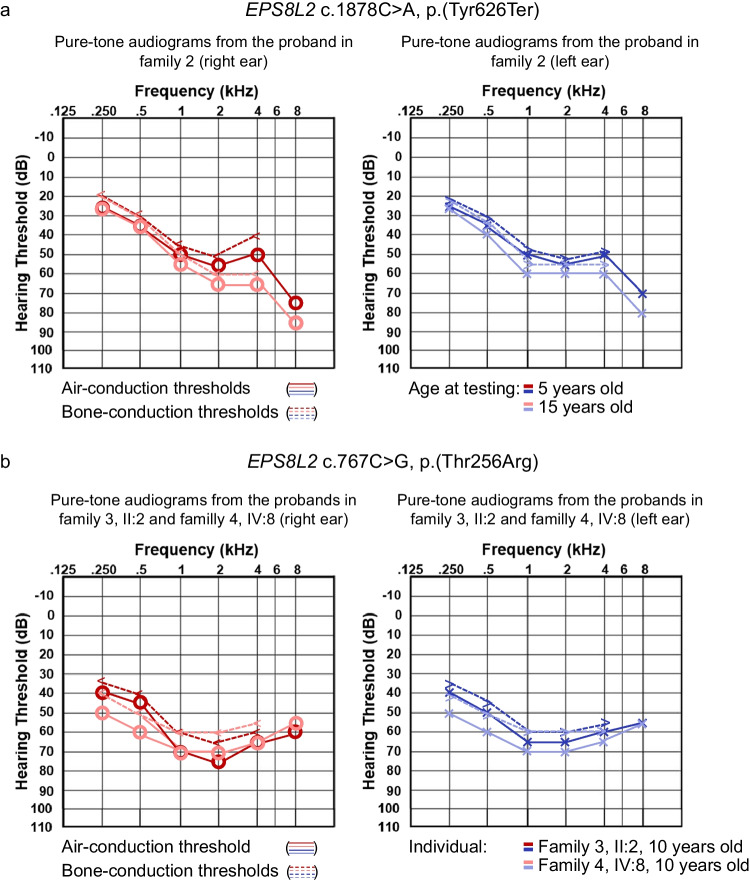
Pure-tone audiograms of probands in Families 2, 3, and 4. **a** A pure-tone audiogram of individual II:1 at the age of 5 and 15 years showed stable sensorineural hearing loss most severe in high frequencies. **b** Pure-tone audiograms of individual II:2 in family 3 and individual IV:8 in family 4 showed similar U-shaped audiograms with hearing most severely affected in the middle frequencies between 1 and 4 kHz. Sensorineural hearing loss is symmetrical and remarkably similar

The family of Fars (Iran) origin shows a history of parental consanguinity but no previous history of hearing impairment (Fig. 1A). This proband was reported previously without describing detailed clinical information [12]. The family recognized hearing loss during registration for preschool when medical examinations that included assessment of hearing were performed and was the original reason for a clinical diagnosis of hearing loss at the time. The proband underwent pure-tone audiometry at the ages of 5 and 15 years, revealing bilateral moderate sensorineural hearing loss with a sloping audiogram profile. Deterioration over this time did not meet the definition of progressive hearing loss (PTA_0.5–4K _47-dB hearing level at the age of 5 years versus PTA_0.5–4K_ 51-dB hearing level at the age of 15 years; change of 4-dB hearing level) (Fig. 2A).

A homozygous c.1878C > A, p.(Tyr626Ter) likely pathogenic variant (PVS1_VS, PM2_P) in exon 19 was identified (Table 1). The nonsense variant occurs in the sterile alpha motif/pointed domain (SAM/PNT) (Fig. 1C). Sanger sequencing of the variant identified both parents as heterozygous carriers and confirmed homozygosity in the proband. The variant is absent from population frequency databases used and is absent from ClinVar and the DVD. This variant was previously published in a large cohort study without any opportunity to show clinical information in detail [12].

**Table 1:** *EPS8L2* variants described in the present study

F	Chr11 Genomic location (g.)	*EPS8L2* c. position	EPS8L2 p. position	Zyg	AF gnomAD (v.4.1.0)	MAF gnomAD (v4.1.0)	MAF Pop gnomAD (v4.1.0)	TOPMed v8	All of Us	SIFT	PP-2	FATHMM	MT	REVEL	CADD
1	721614_721623dup	c.818_827dup	p.Ala279Glyfs*36	Het	NP	NP	NP	NP	NP	NS	NS	NS	NS	NS	NS
1	723329dup	c.1430dup	p.Val478Serfs*25	Het	4.8 e-4	3.8e-4	European (non-Finnish)	1.9e-4	NP	NS	NS	NS	NS	NS	22.8^b^
2	726428C>A	c.1878C>A	p.Tyr626Ter	Hom	NP	NP	NP	NP	NP	NS	NS	NS	NS	NS	36.0^a^
3, 4	721351C>G	c.767C>G	p.Thr256Arg	Hom	NP	NP	NP	NP	NP	D	PrD	T	B	B	31.0

All variants are annotated according to NC_000011.10 (genomic, GRCh38) and NM_022772.4 (coding DNA). Residue position is according to NP_073609.2. *AF* allele frequency, *B* benign, *Chr* chromosome, *D* deleterious, *F* family, *MAF* maximum allele frequency, *MT* MutationTaster, *NP* not present, *NS* not scored, *Pop* population, *PP2* PolyPhen-2, *PrD* probably damaging, *T* tolerated, *Zyg* zygosity. Pathogenicity is represented as ^a^deleterious or ^b^neutral prediction

#### Families 3 and 4

Families 3 and 4 originate from Iran. There is a history of parental consanguinity in two branches of Family 4 (Fig. 1A). Newborn hearing screening was not performed. Pure-tone audiometry from the proband of Family 3 at the age of 7 years showed bilateral moderate sensorineural hearing loss (PTA_0.5–4K_ 63.75- and 60-dB hearing level for the right and left ears, respectively) with a U-shaped audiogram. The parents reported that hearing loss was progressive. However, serial audiograms were unavailable for the quantification of progression. Speech reception thresholds were 60 dB in both ears, with 88% and 96% speech discrimination in the right and left ears, respectively.

Pure-tone audiometry in Family 4 was only available for individual IV:8 at the age of 10 years and showed moderate bilateral sensorineural hearing loss (PTA_0.5–4K_ 65- and 66.25-dB hearing level for the right and left ears, respectively) that was described as progressive. The individual is currently in her fourth decade of life and has described her hearing loss as stable based on the last decade of audiometric follow-up. Speech reception thresholds were 65 and 70 dB for the right and left ears, respectively, and speech discrimination was 70% bilaterally. The hearing loss in both affected individuals is remarkably similar with a relatively flat or gentle U-shaped audiogram profile (Fig. 2B).

A homozygous c.767C > G, p.(Thr256Arg) variant in exon 9 was identified. The variant is absent from gnomAD v4.1.0, as well as the All of Us and TopMed v8 browsers and showed a mixed range of pathogenicity prediction scores (Table 1). Splicing prediction similarly predicted a variable possibility of aberrant splicing (Table 2). Autozygosity analysis of the exome data in all three sequenced affected individuals (Family 3 II:2 and Family 4 IV:7 and IV:8) was performed. Autozygosity mapping uncovered a common run of homozygosity overlapping the genomic coordinates of *EPS8L2*, measuring 4.17 Mb, 5.42 Mb and 20.42 Mb in Family 3 II:2, Family 4 IV:7 and IV:8, respectively (Fig. 1B, Table 3), with an overlap of genomic coordinates Chr11:g.197337–4367675 (GRCh38).

**Table 2:** Splice prediction of the *EPS8L2* c.767C > G, p.Thr256Arg homozygous variant

Variant	SpliceSiteFinder-like	MaxEntScan	NNSPLICE	GeneSplicer	SpliceAI 10 k [≥ 0.2|0.5|0.8]	AbSplice [≥ 0.01|0.05|0.2]
c.767C > G	No change	−34.9%	Not reported	−20.3%	No change	No change

**Table 3:** Shared run of autozygosity on chromosome 11 for the *EPS8L2* c.767C > G, p.Thr256Arg homozygous variant

Individual	Chr11 autozygosity interval	Size (Mb)	*EPS8L2* c.767C > G variant Chr11:g.721351C > G included?
Family 3, Individual II:2	g.197337–4367675	4.17	Yes
Family 4, Individual IV:7	g.87268–5507688	5.42	Yes
Family 4, Individual IV:8	g.192997–20617911	20.42	Yes

Genomic positions are annotated to the human genome GRCh38 assembly

*Mb* megabase

### *In Vitro* Analysis of the *EPS8L2* c.767C > G Variant Shows Aberrant Splicing

**Fig. 3:**
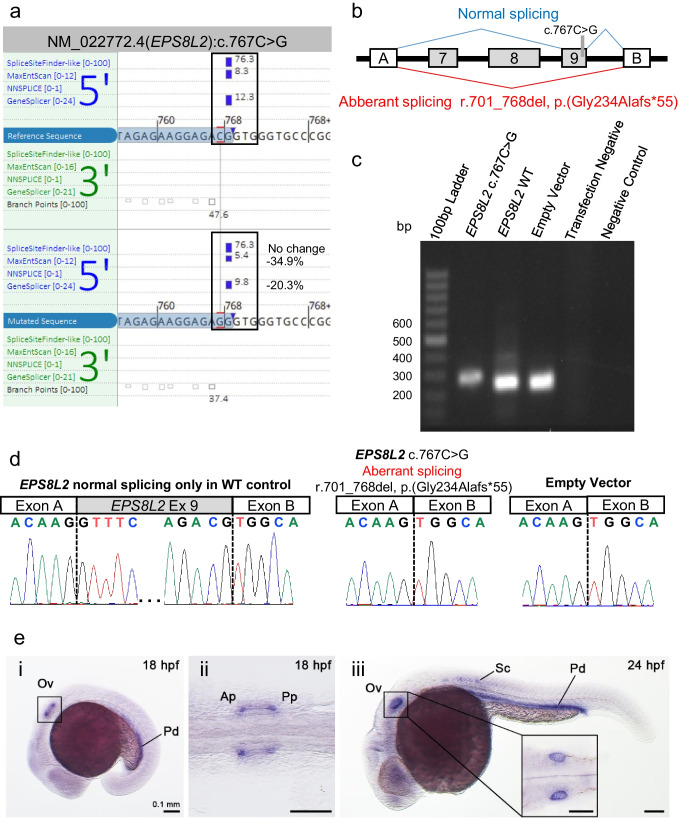
RNA analysis of the *EPS8L2* c.767C > G variant and expression in the developing zebrafish. **a** In silico splice predictions of the wild-type (top, red C) and c.767C > G variant (bottom, red G). The splice scores at the native splice donor site for wild-type and variant are boxed in black. Only scores that are predicted to change are shown. **b** Splicing schematic of the constructs used to test *EPS8L2* c.767C > G variants. The wild-type (upper, blue splice profile) and exon 9 skipping patterns (lower, red splice profile) of the amplicon inserted between exons A and B of the pET01 vector are shown. The *EPS8L2* exons are shown in gray boxes. The gray bar indicates the position of the variant. **c** Gel electrophoresis of the RT-PCR of the *EPS8L2* c.767C > G variant with corresponding wild-type controls, as well as empty pET01 vector amplicons. Transfection negative and PCR negative controls performed as expected. **d** Sanger sequencing of exon-exon junctions. The *EPS8L2* c.767C > G variant causes aberrant skipping (middle panel) resembling the empty vector control (right panel), while the wild-type control also shows normal splicing (left panel). **e** *eps8l2* is expressed in the developing inner ear and pronephros in zebrafish. (i) At 18 hpf, *eps8l2* mRNA is expressed in the otic vesicles (Ov) and pronephric duct (Pd). (ii) In the enlarged otic vesicle (indicated by the black empty box), *eps8l2* expression is abundant at both the anterior and posterior poles. Ap, anterior pole. Pp, posterior pole. (iii) At 24 hpf, *eps8l2* mRNA is persistently expressed in the otic vesicle and pronephric duct, with scattered expression in the spinal cord (Sc). Zebrafish head faces to the left and dorsal to the top. Scale bar = 0.1 mm

*In silico* splice predictions of the *EPS8L2* c.767C > G variant showed reductions in the splice site donor scores of exon 9 for MaxEntScan and GeneSplicer scores, while the other four tools used (SpliceSiteFinder-like, NNSPLICE, SpliceAI, and AbSplice) did not show a change in the reference splice score (Fig. 3A, Table 2). Nonetheless, an *in vitro* splice (minigene) assay was performed and RT-PCR including the target exon 9 in the wild-type (WT) control showed amplicons with a mixture of normal splicing pattern and exon skipping (Fig. 3B) that was further analyzed with TOPO cloning and confirmed by Sanger sequencing. Amplicons from the *EPS8L2* c.767C > G construct showed complete exon 9 skipping r.701_768del, p.(Gly234Alafs*55) confirmed by Sanger sequencing (Fig. 3B–D). The variant was classified as likely pathogenic (PM2_P, PM3_M, PVS1_S).

### *eps8l2* mRNA Expressed in the Developing Inner Ear and Pronephros During Zebrafish Development

We aimed to examine the expression pattern of *eps8l2 *mRNA during zebrafish development to better understand its potential role in hearing. WISH revealed that *eps8l2 *is expressed in the otic vesicles, specifically at the anterior (presumptive utricular macula) and posterior (presumptive saccular macula) poles, as well as in the pronephric duct (Fig. 3E (i–ii)). At 24 h post-fertilization (hpf), as the otic vesicles undergo significant morphogenesis, *eps8l2 *remains ubiquitously expressed throughout the otic vesicles and pronephric duct with additional scattered expression in the spinal cord (Fig. 3E (iii)).

## Discussion

Hearing and balance rely on the conversion of mechanical stimuli into electrical signals, facilitated by mechanically gated ion channels in the stereocilia of sensory hair cells. These stereocilia, supported by actin filaments, form hair bundles whose size and structure vary by location and undergo precise regulation during development and maintenance [13]. This process involves actin-binding proteins and myosin motors, but the molecular mechanisms remain incompletely understood [4]. Previous research highlights the role of Eps8 and its related protein Eps8l2 in stereociliary growth [4]. Analysis of the *Eps8l2 *knockout mouse showed that the absence leads to progressive hearing loss due to disorganized hair bundles with variations in the length and width of stereocilia and is required for the maintenance and function of mechanosensory hair bundles [4].

We identified two variants c.818_827dup, p.(Ala279Glyfs*36) and c.1430dup, p.(Val478Serfs*25) resulting in a frameshift, the first reported nonsense variant c.1878C > A, p.(Tyr626Ter), and the first missense variant with spliceogenic effect c.767C > G, p.(Thr256Arg). *In vitro* analysis of the *EPS8L2* c.767C > G variant demonstrates loss of function effects through skipping of exon 9, leading to a frameshift deletion r.701_768del, p.(Gly234Alafs*55). Only two out of six total *in silico* splice prediction tools predicted aberrant splicing (Table 2). This emphasizes the importance of testing RNA-level changes on variants with limited *in silico* evidence, as splice predictors must also be evaluated on a gene-by-gene basis to identify tools with highest prediction accuracy and this information is lacking for the majority of disease-associated genes [14]. Aberrantly spliced amplicons in the WT construct are attributed to alternative splicing; however, the frameshift due to aberrant splicing of the c.767C > G variant would impact a biologically relevant exon, reinforced by a previously described pathogenic c.738del, p.(Val247Cysfs*6) variant that also causes a frameshift due to a deletion in exon 9 [9]. All variants are predicted to lead to a premature stop codon likely to trigger nonsense-mediated decay (NMD). NMD machinery differentiates between a regular stop codon and premature termination codons (PTCs) by assessing the position of the PTC within newly synthesized mRNA. When a PTC resides at least 50–55 bp upstream of the 3′-most exon-exon junction, the NMD pathway is activated to degrade unfavorable transcripts [15]. In the case of the novel c.1878C > A, p.(Tyr626Ter) variant, the PTC is in exon 19 out of 21 exons, making NMD activation likely. All previously reported variants were deletion, insertion, or duplication variants (Table 4). This study serves as a valuable expansion to the mutational landscape of *EPS8L2*-associated hearing loss.

**Table 4:** Summary of families with *EPS8L2* (NM_022772.4) associated hearing impairment

ID	*EPS8L2* c. position	EPS8L2 p. position	Exon	Age of onset	Severity	Progression	Self-described ethnicity
Family 1, II:1 current study	c.818_827dup c.1430dup	p.(Ala279Glyfs*36) p.(Val478Serfs*25)	10 15	4 years	Moderate-severe	Yes	German
Family 1, II:2 current study	c.818_827dup c.1430dup	p.(Ala279Glyfs*36) p.(Val478Serfs*25)	10 15	Observed by parents first at 23 months; confirmed at 2.5 years	ND	ND	German
Family 2, II:1 current study (Alerasool et al., 2024)	c.1878C > A	p.(Tyr626Ter)	19	Preschool age	Moderate	No	Iranian
Family 3, II:2 current study	c.767C > G	p.(Thr256Arg)	9	7 years	Moderate	Yes	Iranian
Family 4, IV:8 current study	c.767C > G	p.(Thr256Arg)	9	Prelingual	Moderate	Yes	Iranian
Dahmani et al., 2015	c.1014delC	p.(Ser339Alafs*15)	12	~ 4 years	Sloping audiogram, high-frequencies severely affected	Yes	Algerian
Wang et al., 2017	c.738delC	p.(Val247Cysfs*6)	9	ND	Moderate	ND	Pakistani
Abu Rayyan et al., 2020	c.148_159insGGACA	p.(Ser50Trpfs*34)	4	Prelingual	Profound	ND	Palestinian
Abu Rayyan et al., 2020	c.1430dup	p.(Val478Serfs*25)	15	~ 6 years	Profound	Yes	Palestinian

*ND* not described

The c.767C > G, p.(Thr256Arg) variant in Families 3 and 4 exhibited a cryptic splice effect r.701_768del, p.(Gly234Alafs*55). Both families originated from Iran but were recruited by independent collaborating teams. Autozygosity mapping identified a shared 4.17 Mb run of homozygosity, suggesting a potential founder effect. In the UCL Queen Square Genomics internal database (~ 40,000 exomes), the *EPS8L2* (NM_022772.4):c.767C > G, p.(Thr256Arg) variant was observed in one carrier only. Furthermore, this variant was absent from gnomAD, TopMed, and the All of Us databases. It was also not detected in Iranome, a population-specific database, though this may in part reflect the limited sequencing coverage in underrepresented populations and the great diversity of sub-populations in Iran.

So far, four families have been reported in the literature (Table 4) and we present four additional families that further define the natural history of DFNB106-associated hearing impairment. The age of onset was described as prelingual/preschool age in 5 out of 7 individuals from whom information was available and around 6 and 7 years of age for two others. Hearing loss was recorded as moderate in half of the individuals, and thresholds reached severe to profound levels in the other half. Interestingly, it is progressive in five out of six individuals in whom this was traced. Progressive hearing loss is rather rare for autosomal recessive non-syndromic hearing loss [11]. We also show a unique U-shaped audiogram, which has not been previously reported for *EPS8L2*-associated hearing impairment in two individuals with the spliceogenic c.767C > G, p.(Thr256Arg) variant with a molecular effect r.701_768del, p.(Gly234Alafs*55). Importantly, longitudinal follow-up of the younger sibling of the proband in Family 1, who had passed newborn hearing screening despite being compound heterozygous for the same *EPS8L2* variants, revealed the onset of hearing loss at approximately 23 months of age. This child represents the first documented case of *EPS8L2*-associated hearing loss in a European family and provides the first evidence of a pre-symptomatic individual developing measurable hearing loss after an initially normal screening result. This finding reinforces the delayed-onset and progressive nature of *EPS8L2*-related hearing loss and underscores the importance of early molecular diagnosis, close audiological monitoring, and timely genetic referral. This highlights a potential therapeutic window and the need for early referral for genetic testing. Continued clinical follow-up in otolaryngology is recommended. All individuals who we present show moderate/moderate-severe hearing impairment.

To date, there have been no preclinical therapy studies for DFNB106 using the available *Eps8l2* knockout or newly generated mouse models [4]. The *Eps8l2* knockout model shows a late onset, progressive hearing loss that potentially mirrors the clinical situation in patients, at least to some extent, and serves as a good model for preclinical testing. A preclinical gene therapy study of an *Eps8* knockout mouse using adeno-associated virus (AAV) delivery of the *EPS8* transgene with the Anc80L65 AAV serotype showed rescue to the hair bundle structure but failed to rescue functional hearing [16] and achieved a 94% and 44% transduction rate of apical IHCs and OHCs, respectively. When delivered in *Eps8* knockout mice at postnatal days 1 and 2, stereocilia growth was variably improved, with expression restored to the tips of the stereocilia. When delayed to postnatal day 3, almost no recovery was observed, shrinking the targeted delivery of all studies to between 1 and 2 days after birth. Interestingly, mechanoelectrical transduction was rescued in treated mice but attachment to the tectorial membrane was lost and hearing recovery was not achieved [16]. However, the developmental roles of EPS8 and EPS8L2 may be different, using hearing loss onset between the two knockout mouse studies as a proxy [4, 6].

In summary, this work doubles the number of reported families with *EPS8L2*-associated hearing impairment. Importantly, it shows a potential therapeutic window by confirming that normal hearing at birth is possible. *EPS8L2* is a unique form of hearing loss in that it has a progressive character in most individuals reported, and individuals can present with a U-shaped audiogram. However, additional case series are essential for understanding the prevalence and rate of progression for optimizing future therapeutic opportunities. An early molecular genetic diagnosis is crucial for optimizing hearing outcomes. This is potentially even more so true for *EPS8L2* due to its delayed onset and progressive character.

## Materials and Methods

### Patient Recruitment and Clinical Assessment

We evaluated two non-consanguineous and two consanguineous families. Three families (one non-consanguineous and two consanguineous) from Iran were identified through a large ethnically diverse Iranian population rare disease study consisting of approximately 800 probands with the sole inclusion criteria being hereditary hearing impairment and data sharing with colleagues. One additional non-consanguineous family was recruited in Germany. This study was approved by the Medical Faculty of the University of Würzburg, Germany (approval number 46/15), and the Ethics Committee of Mashhad University of Medical Sciences (IR.MUMS.MEDICAL.REC.1402.090). Blood samples were collected after obtaining informed consent from patients or their parents. Written informed consent from the parents or legal guardians of the patients/participants was obtained for the publication of their data.

Demographic, otolaryngologic, audiological, and relevant medical data were ascertained from the medical records of probands (Family 1 II:1, Family 2 II:1, Family 3 II:2, and Family 4 IV:7 and IV:8). Affected individuals underwent a complete otologic evaluation. Routine pure-tone audiometry was performed according to current standards and measured hearing thresholds at 0.25, 0.5, 1, 2, 4, and 8 kHz. Air- and bone-conduction thresholds were measured, and severity of hearing loss was determined by averaging pure-tone thresholds over 0.5, 1, 2, and 4 kHz (pure-tone average, PTA_0.5–4K_). The severity of hearing loss in the better ear was defined as mild for thresholds averaging 20–40-dB hearing level, moderate for 41–70-dB hearing level, severe for 71–95-dB hearing level, and profound in excess of 95-dB hearing level. Progressive hearing loss was defined as a deterioration of > 15-dB hearing level in the average over the frequencies of 0.5, 1, and 2 kHz within a 10-year period.

### Exome Sequencing, Bioinformatics, and Variant Classification

Exome and Sanger sequencing were performed independently. The proband of each family was selected for exome sequencing. Initially, Family 4 was recruited as two separate families, but later, upon detailed review, a common ancestor was disclosed. Thus, the family was revised as a single large family with two extended branches of individuals with non-syndromic hearing loss. In this case, we sequenced two affected individuals from Family 4 (IV:7 and IV:8) due to the original classification of two families.

Following extraction of DNAs from whole blood by standard protocols, proband DNA samples were subjected to exome capture using the Agilent SureSelect Human All Exon V6 Kit (Agilent, Santa Clara, CA, USA) (Family 1 II:1 and Family 2 II:1) or the Nextera DNA Exome (Illumina, Inc., San Diego, CA, USA) (Family 3 II:2 and Family 4 IV:7 and IV:8) according to manufacturer’s protocols and paired-end sequenced on an Illumina sequencer. The proband of Family 1 (II:1) was sequenced and analyzed at the Institute of Human Genetics at the University Medical Centre of the Johannes Gutenberg University Mainz as previously described [17]. The proband of Family 2 (II:1) was referred externally for exome sequencing and processed as previously described [12]. Individual II:2 from Family 3 and IV:7 and IV:8 from Family 4 were sequenced at the Institute of Human Genetics at the University of Würzburg, Germany, and analyzed using GensearchNGS software (PhenoSystems SA, Wallonia, Belgium). Copy number variation analysis was performed. A focus on hearing loss–associated genes was prioritized in exome analyses done at all centers.

The generated sequences were demultiplexed and mapped to the human genome reference (GRCh38) with Burrows Wheeler Aligner for subsequent variant calling. Bioinformatics filtering strategy focused on exonic and donor/acceptor splicing variants. Alternative alleles present at > 20% and a minor allele frequency < 0.01 were assessed using gnomAD [18], TopMed [19], and the All of Us research program [20]. *In silico* pathogenicity predictions applied SIFT [21], PolyPhen-2 [22], FATHMM [23], MutationTaster [24], REVEL [25], and CADD [26]. Computational assessment of splicing effects used SpliceSiteFinder-like, MaxEntScan, NNSplice, and GeneSplicer embedded in Alamut Visual Plus v1.12 (Sophia Genetics, Bidart, France), as well as SpliceAI Visual and AbSplice embedded in SpliceAI Visual [27].

Pathogenicity of detected variants was evaluated according to the hearing loss–adapted American College of Medical Genetics/Association for Molecular Pathology (ACMG/AMP) guidelines [28] and updated PVS1 guidelines [29] applied to *EPS8L2* with a current moderate gene-disease validity classification. Variants were analyzed in ClinVar and the DVD [30] to assess previous variant classifications. Detected variants were subjected to segregation analysis by Sanger sequencing with the DNA samples from available family members. Primers are shown in Supplementary Table 1. Visualization of truncated variants and variant nomenclature validation was performed using Mutalyzer [31].

### Autozygosity Mapping

The three affected individuals who were exome sequenced in Family 3 (II:2) and Family 4 (IV:7 and IV:8) presented the same homozygous *EPS8L2* missense variant c.767C > G, p.(Thr256Arg). Shared regions of homozygosity were assessed by importing VCF files into AutoMap software [32].

### *In Vitro* Splice Assay

Computational assessment of splicing effects used SpliceSiteFinder-like, MaxEntScan, NNSplice, and GeneSplicer embedded in Alamut Visual Plus v1.12 (Sophia Genetics, Bidart, France), as well as SpliceAI 10K and AbSplice [33] as included in SpliceAI Visual [27].

RNA studies of variants were conducted following established protocols with modifications [34] using constructs annotated to NM_022772.4. In brief, the minigene construct comprised a 761-bp region spanning from 98 bp before exon 7 to 140 bp after exon 9, encompassing the *EPS8L2* c.767C variant region. This region was amplified from a healthy control using primers containing specific restriction sites. All primers are shown in Supplementary Table 1. The PCR fragments were ligated between exons A and B of the linearized pET01 vector following digestion with restriction enzymes. The recombinant vectors were transformed into DH5α competent cells (NEB 5-alpha, New England Biolabs, Frankfurt, Germany), plated, and incubated overnight. After conducting colony PCR with pET01 PCR Primer 02 F and the cloning target-specific reverse primer, the mutant-containing vector was generated using site-directed mutagenesis-specific primers. The sequences of both the wild-type and mutant-containing vectors were confirmed through Sanger sequencing. Subsequently, these vectors were transfected into HCT116 cells (ATCC, Manassas, VA, USA). Transfection was achieved by introducing 2 µg of the respective pET01 vectors using 6 µL of FuGENE 6 Transfection Reagent (Promega, Walldorf, Germany). The empty vector reaction was also included in the experiment as a control. The cells were then harvested 48 h post-transfection, and total RNA was isolated using the miRNeasy Mini Kit (Qiagen, Hilden, Germany). cDNA was synthesized using the High Capacity cDNA Reverse Transcription Kit (Applied Biosystems, Waltham, MA, USA) and pET01 cDNA primer 01 following the manufacturer’s protocols. cDNA was PCR amplified using vector-exon specific primers. The amplified fragments were visualized on a 1% agarose gel. cDNA amplicons of the WT construct were TOPO cloned following standard protocols with the Zero Blunt TOPO PCR Cloning Kit (ThermoFisher, Darmstadt, Germany) and Sanger sequenced.

### Zebrafish Husbandry

Zebrafish experiments were conducted in the NHGRI-1 wild-type strain in adherence to the Institutional Animal Care Committee (IACUC) of the Oklahoma Medical Research Foundation (22-18) approved protocol. All animals were reared and kept under standard conditions in an Association for Assessment and Accreditation of Laboratory Animal Care (AAALAC) accredited facility.

### Whole-Mount *In Situ* Hybridization (WISH)

A previously described technique [35] was used to perform whole-mount *in situ* hybridization (WISH). Briefly, a 784-bp fragment was PCR-amplified from zebrafish cDNA using primers carrying the T3 promoter sequence at the 5′ end of the *eps8l2*-specific forward primer and the T7 promoter sequence at the 5′ end of the reverse primer (Supplementary Table 1). The amplified DNA fragments were used to synthesize an antisense riboprobe labeled with digoxigenin-UTP. Wild-type embryos at the 18- and 24-h post-fertilization (hpf) stage were treated with 0.003% 1-phenyl-2-thiourea (Millipore Sigma, MO, USA) to prevent pigmentation. Embryos were fixed with 4% (V/V) paraformaldehyde and dehydrated through increasing methanol concentrations before storage in 100% methanol at −20 °C overnight. The following day, embryos were rehydrated using decreasing methanol concentrations and permeabilized with 10 µg/mL proteinase K (Invitrogen). Color development was performed using BM purple alkaline phosphatase substrate (Millipore Sigma, MO, USA).

## Supplementary Information

Below is the link to the electronic supplementary material.

ESM 1 (PDF 229 KB)

ESM 2 (PDF 20.1 KB)

## Data Availability

The datasets generated and/or analysed during the current study are not publicly available due to privacy restrictions but can be made available from the corresponding author on reasonable request. All variants have been submitted to ClinVar under accession ID SUB15530400.
