# Supplementary material for: Expansion of Molecular and Clinical Aspects of EPS8L2 (DFNB106)-Associated Hearing Loss Emphasizes a Potential Therapeutic Window
Source: Mol Neurobiol. 2026 Jan 10;63(1):354. doi: 10.1007/s12035-025-05615-9 (PMC12789209; doi:10.1007/s12035-025-05615-9)

Full unedited gel for EPS8L2 minigene result:

Lane 1: empty

Lane 2: empty

Lane 3: empty

Lane 4: **100 bp ladder**

Lane 5: ***EPS8L2* c.767C>G**

Lane 6: ***EPS8L2* WT**

Lane 7: **Empty vector**

Lane 8: **Transfection Control**

Lane 9: **Negative Control**

Lane 10: empty

Lane 11: empty

Lane 12: empty

Lane 13: empty

Lane 14: empty

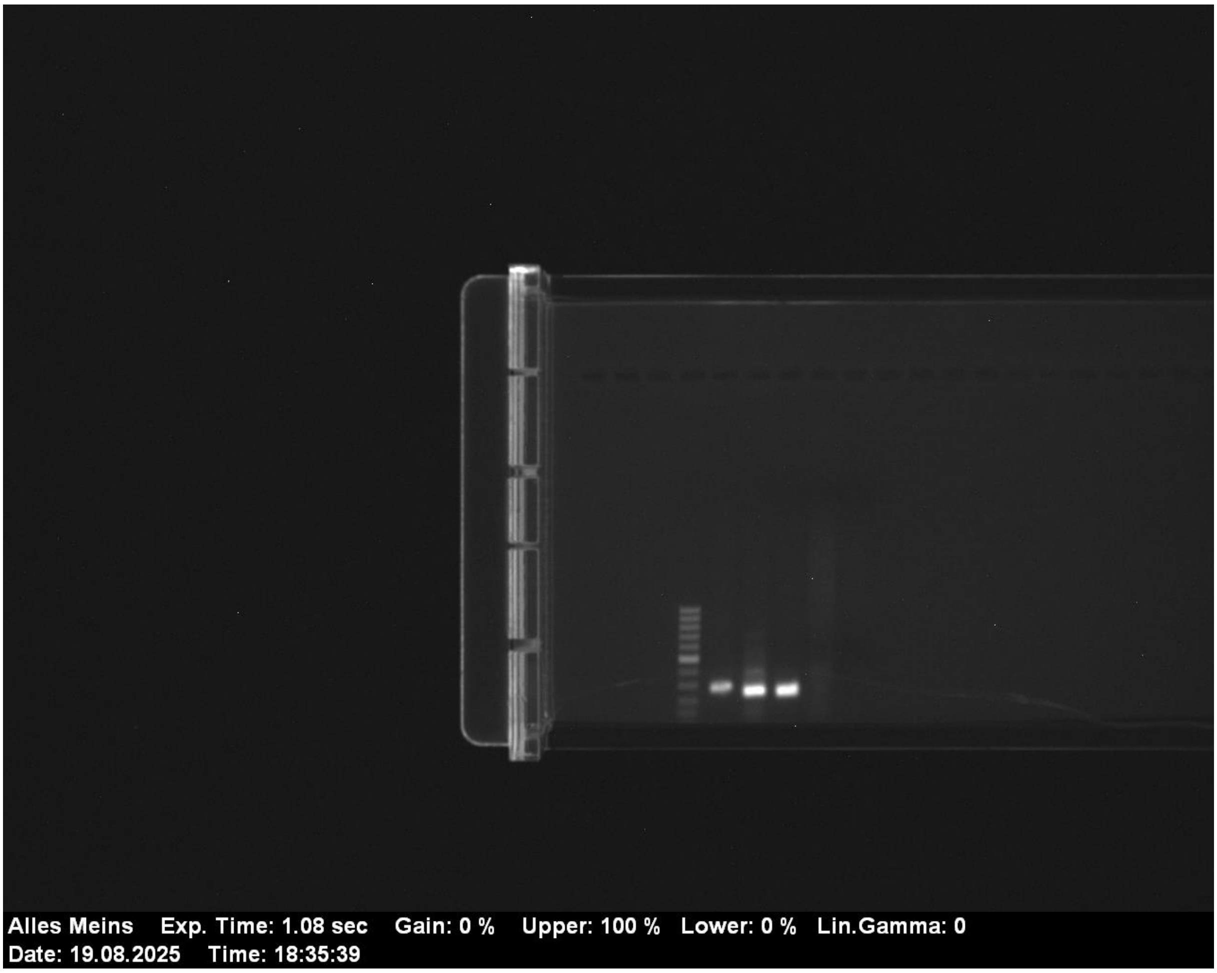

Supplement: Supplementary file 1 — (PDF 229 KB) [file 12035_2025_5615_MOESM1_ESM.pdf]
