# Supplementary material for: Expansion of Molecular and Clinical Aspects of EPS8L2 (DFNB106)-Associated Hearing Loss Emphasizes a Potential Therapeutic Window
Source: Mol Neurobiol. 2026 Jan 10;63(1):354. doi: 10.1007/s12035-025-05615-9 (PMC12789209; doi:10.1007/s12035-025-05615-9)
Supplement: Supplementary file 2 — (PDF 20.1 KB) [file 12035_2025_5615_MOESM2_ESM.pdf]

Supplementary Table 1 Primers used in segregation analysis and splice assay

Primer	Primer Name	Primer Sequence 5´- 3´	Product Size
EPS8L2 Ex9 segregation	*EPS8L2* Ex9 F	GGGACACCAGGAGAAGATTC	363 bp
	EPS8L2 Ex9 R	AGGGACAGCAAGGACCAGT	
Cloning Primers	hu_*EPS8L2*_Ex7-9_XhoI_F	aattctcgagTGCGATGAGGTGGAGGTGAG	761 bp
	hu_*EPS8L2*_Ex7-9_BamHI_R	attggatccACAGATGATGGGAGGAGCTG	
EPS8L2 c.767C-G Mut	*EPS8L2*_c.767C-G_mut_F	GAGAAGGAGAgGGTGGGTGCC	4.9 kb
	*EPS8L2*_c.767C-G_mut_R	TATCTTCTGAGCCAGCACG	
cDNA Synthesis	pET01 cDNA primer 01	GATCCACGATGC	NA
pET01 exons A and B	pET01 PCR primer 02 F	GATGGATCCGCTTCCTGCCCC	314 bp/ 246 bp
	pET01 PCR primer 03 R	CTCCCGGGCCACCTCCAGTGCC	
TOPO Cloning	M13 forward	GTAAAACGACGGCCAG	514 bp/ 446 bp
	M13 reverse	CAGGAAACAGCTATGACC	
Whole-mount in situ hybridization	eps8l2 forward	gaattgaattaaccctcactaaagggGCTAACTCTACGAGCTAAGCC	NA
	eps8l2 reverse	gaattgtaatacgactcactatagggGTCTTTATCACTGCCCTGCG	
